# Theoretical Photoelectron Spectroscopy of Low-Valent Carbon Species: A ∼6 eV Range of Ionization Potentials among Carbenes, Ylides, and Carbodiphosphoranes

**DOI:** 10.1021/acsorginorgau.2c00045

**Published:** 2022-12-02

**Authors:** Abhik Ghosh, Jeanet Conradie

**Affiliations:** † Department of Chemistry, University of Tromsø, Tromsø N-9037, Norway; ‡ Department of Chemistry, University of the Free State, Bloemfontein 9300, Republic of South Africa

**Keywords:** carbene, ylide, carbodiphosphorane, carbodicarbene, carbone

## Abstract

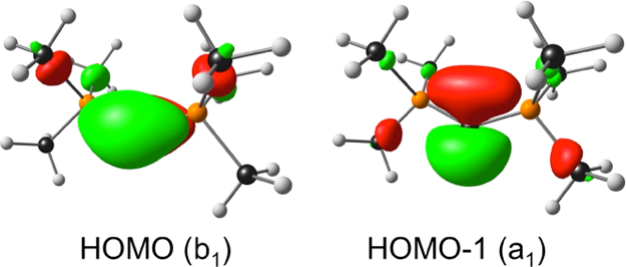

High-quality density functional theory calculations underscore a nearly 6 eV range for the ionization potentials (IPs) of neutral, low-valent carbon compounds, including carbenes, ylides, and zero-valent carbon compounds (carbones) such as carbodiphosphoranes (CDPs) and carbodicarbenes. Thus, adiabatic IPs as low as 5.5 ± 0.1 eV are predicted for CDPs, which are about 0.7–1.2 eV lower than those of simple phosphorus and sulfur ylides. In contrast, the corresponding values for *N*-heterocyclic carbenes are about 8.0 eV while those for simple singlet carbenes such as dichlorocarbene and difluorocarbene range from about 9.0 eV to well over 11.0 eV.

## Introduction

Core ionization potentials (IPs), as measured by X-ray photoelectron spectroscopy, provide exquisitely sensitive measures of local electrostatic potential and thereby of the oxidation state and substituent effects.^1−3^ Valence IPs, for highly localized orbitals, in principle can provide similar information. Classic singlet carbenes^4−6^ and stabilized Arduengo-type nucleophilic carbenes,^7−9^ with localized carbon lone pairs, are understandably attractive targets for gas-phase photoelectron spectroscopy (PES). Thus, difluorocarbene,^10,11^ dichlorocarbene,^12,13^ and an *N*,*N*′-dialkylimidazol-2-ylidene^14^ have all been studied, and the IPs for the carbene lone pair have been found to be 11.37, 9.27, and 7.68 eV, respectively. Moreover, in our early work on PES on porphyrins^15−20^ (and other recent work^21,22^), we established that density functional theory (DFT) calculations are able to reproduce the lowest gas-phase IPs with near-quantitative accuracy. The latter finding has encouraged us to conduct a theoretical survey of key low-valent carbon species with emphasis on species that have yet to be experimentally studied with gas-phase PES.

Our aim here has been to determine the lowest IPs of carbodiphosphoranes (CDPs), which resemble carbenes in being dicoordinate but, unlike carbenes (which contain divalent carbon), feature *zero-valent* carbon.^23,24^ The CDP carbon accordingly has two lone pairs and a formal charge of −2, as depicted in the general formula R_3_P^+^–C^2–^–P^+^R_3_. The valence of 0 follows from the rule “valence = no. of bonds + formal charge”, that is, 2 + (−2) = 0.^25^ Although the first CDP was reported over half a century ago, the CDP field has recently undergone a renaissance.^26^ Thus, CDPs have been recognized as superbases,^27,28^ as superbase catalysts,^29^ and as potentially doubly dative ligands toward main-group,^30−33^ d-block,^34^ and f-block^35,36^ complexes. Recently, a second class of zero-valent carbon compounds (also called carbones) has emerged—the carbodicarbenes (CDCs)^37−40^—in which nucleophilic carbenes take the place of phosphines in CDPs. As of today, no CDP or CDC has been examined with gas-phase PES. Accordingly, the present calculations include three CDPs and one CDC, and the resulting IPs are viewed against a backdrop of experimental and DFT results for classic carbenes and ylides.

## Results and Discussion

**Figure 1:**
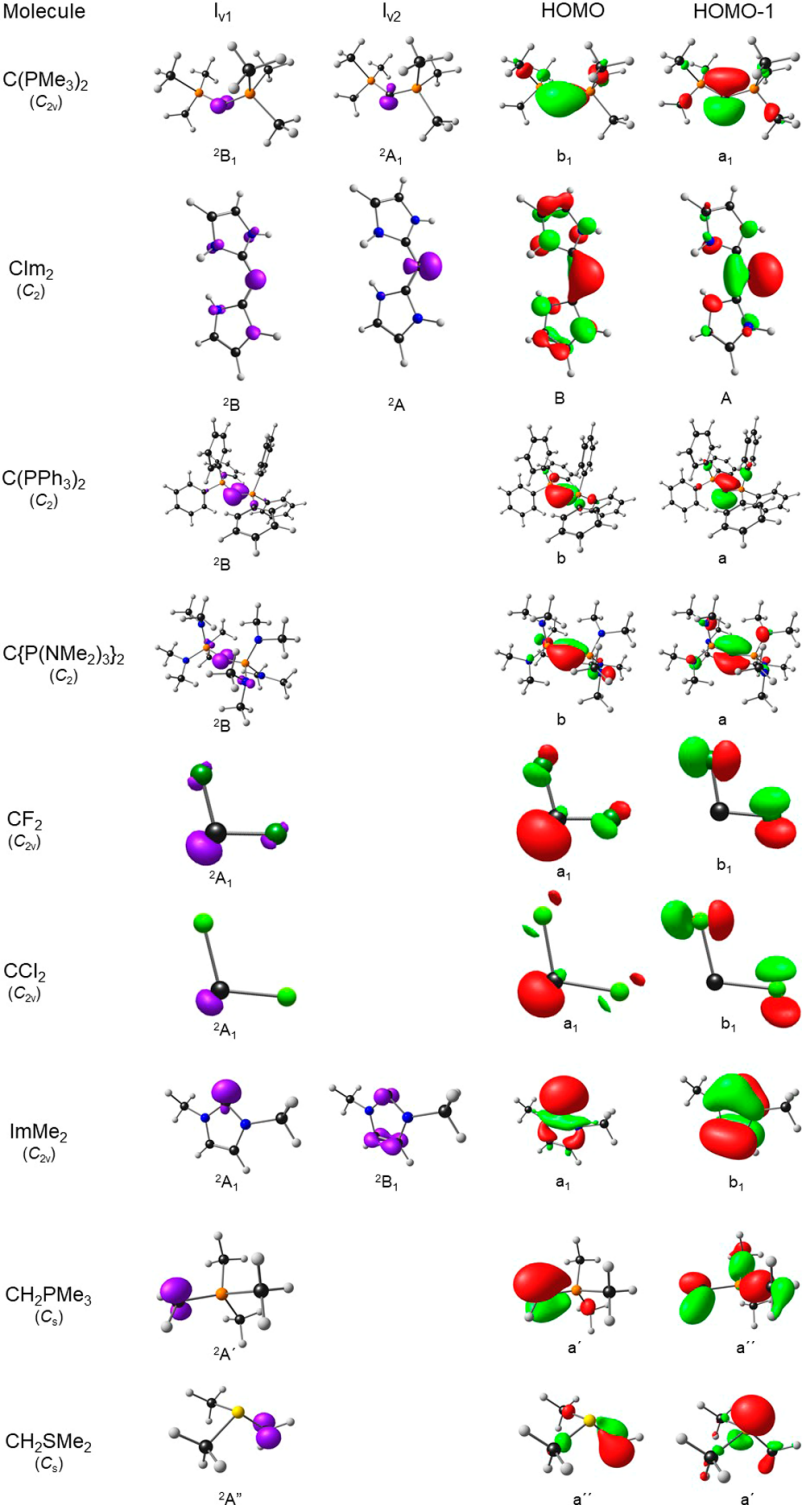
Selected B3LYP-D3/STO-TZ2P results in the graphical form: spin density plots of the lowest (and in two cases the second-lowest) vertically ionized states and the HOMO and HOMO – 1 of the neutral molecules.

Table 1 lists vertical and adiabatic scalar-relativistic^41^ DFT IPs calculated with two different exchange–correlation functionals, OLYP^42,43^ and B3LYP,^44,45^ each augmented with D3^46^ dispersion corrections, and scalar-relativistic all-electron ZORA STO-TZ2P basis sets, all as implemented in the ADF 2019 program system.^47^ Point-group symmetry was used as appropriate (while all structures were confirmed as minima via frequency analyses). Figure 1 depicts the relevant molecular orbitals (MOs) and spin density plots for key ionized states. For purposes of discussion, dichlorocarbene, the first singlet carbene to be studied in detail,^13^ provides an apt starting point for our discussion. As shown in Table 1, a B3LYP-D3 adiabatic IP of 8.98 eV has been calculated for CCl_2_, in rather good agreement with the corresponding experimental value (9.27 eV).^12^ The stabilized, nucleophilic carbene *N*,*N*′-dimethylimidazol-2-ylidene has a significantly lower, calculated B3LYP vertical IP of 7.99 eV for the carbene lone pair; the slightly higher second IP of 8.42 eV corresponds to ionization of the imidazole π-system. These too are in good agreement with experimental PES values of 7.68 and 8.22 eV, respectively, measured for *N*,*N*′-di(^*t*^butyl)imidazole-2-ylidene.^14^ In contrast, a dramatically higher adiabatic IP of 11.26 eV has been calculated for difluorocarbene, again in essentially perfect agreement with the experimental value,^10^ underscoring the powerful electron-withdrawing effect of the two fluorines.

**Table 1:** DFT Calculations of Ionization Potentials for Selected Low-Valent Carbon Species

		OLYP-D3			B3LYP-D3		
molecule	Pt. group	IP_1_	IP_2_	adiabatic IP	IP_1_	IP_2_	adiabatic IP
C(PMe_3_)_2_	*C*_2v_	6.20	6.47	5.84	6.22	6.35	5.84
C(PPh_3_)_2_	*C*_2_	5.92		5.59	5.98		5.57
C{P(NMe_2_)_3_}_2_	*C*_1_	5.85		5.33	6.03		5.44
CIm_2_	*C*_2_	5.46	6.09	5.30	5.55	6.12	5.39
Me_2_Im	*C*_2v_	7.87	8.29	7.46	7.99	8.42	8.10
CF_2_	*C*_2v_	11.82	15.83	11.02	12.11	16.31	11.26
CCl_2_	*C*_2v_	9.82	11.47	8.82	9.92	11.81	8.98
CH_2_PMe_3_	*C*_s_	6.66		6.23	6.68		6.27
CH_2_SMe_2_	*C*_s_	7.02		6.72	7.02		6.71

**Figure 2:**
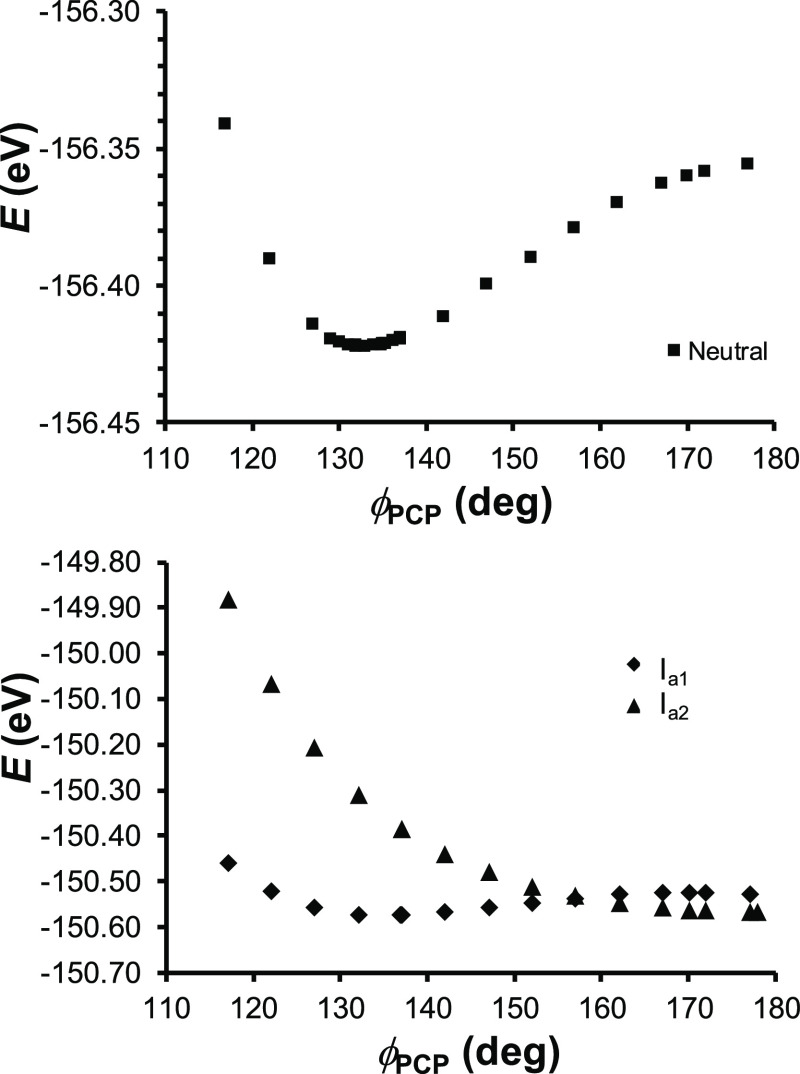
B3LYP-D3/STO-TZ2P potential energy surfaces of neutral (above) and two lowest adiabatic cationic states, I_a1_ (^2^B_1_) and I_a2_ (^2^A_1_), of C(PMe_3_)_2_.

In contrast to the above, the lowest B3LYP-D3 vertical IP of hexamethyl-CDP, C(PMe_3_)_2_, is found to be 6.22 eV, corresponding to ionization of a π (i.e., b_1_ under *C*_2v_) lone pair—approximately 3 eV lower than that of dichlorocarbene. Ionization of the σ lone pair corresponds to a slightly higher IP of 6.35 eV. A substantial relaxation energy is associated with these ionizations; the lowest adiabatic IP (B3LYP-D3) is found to be 5.84, about 0.4 eV lower than the vertical value, suggesting a significant geometrical change upon ionization. As in an earlier study of C(PPh_3_)_2_ by Quinlivan et al.,^31^ our DFT calculations confirm a bent minimum for C(PMe_3_)_2_ with a PCP angle of 156.4° (Figure 2). However, linearization is energetically cheap, costing no more than ∼1 kcal/mol. The calculated potential energy surfaces are even flatter for the two lowest cationic states of C(PMe_3_)_2_.

For the more electron-rich hexaphenyl-CDP^21^ and hexakis(dimethylamino)-CDP,^48^ B3LYP-D3 calculations predict even lower vertical IPs of about 6.0 eV or adiabatic IPs of around 5.5 ± 0.1 eV. The very lowest IP, a B3LYP-D3 adiabatic value of 5.39 eV, has been found for the CDC carbodi(imidazole-2-ylidene), CIm_2_ (Table 1). To help contextualize these values, they are even lower than those calculated for extended π-systems such as simple porphyrins and corroles (∼6.5 eV), which yield air-stable π-cation radicals.^16−20^

The calculated IPs of the CDP and CDC derivatives are also lower than those experimentally observed for ylides such as methylenetrimethylphosphorane, CH_2_PMe_3_^49^ and CH_2_SF_4_,^50^ which are zwitterionic, divalent carbon species with a formal charge of −1 on the ylidic carbon. In the case of CH_2_PMe_3_, the vertical B3LYP-D3 value is in excellent agreement (6.68 eV) with the He I PES value (6.81 eV). The calculated (B3LYP-D3, vertical) IP of CH_2_SMe_2_ (7.02 eV) is somewhat higher, but as far as we have been able to determine, an experimental value is not available for comparison. In contrast, the experimentally reported IP of CH_2_SF_4_ (10.65 eV) is very much higher, reflecting the extreme electron-withdrawing character of the cationic SF_4_^+^ substituent. Qualitatively, these trends in IPs “make sense”, given that CDPs are double ylides and are expected to be more electron-rich than regular ylides.

## Conclusions

DFT calculations predict a nearly 6 eV range for IPs for low-valent carbon compounds, from well under 6.0 eV for the adiabatic IPs of CDPs and CDCs to above 11.0 eV for difluorocarbene. We harbor the hope that the “theoretical photoelectron spectra” predicted here will soon be experimentally confirmed and that the IPs will serve as useful correlates for chemically interesting properties such as basicity, nucleophilicity, and the ability to act as ligands toward a variety of elements.

## Computational Methods

DFT calculations were carried out with two different exchange–correlation functionals, OLYP^40,41^ and B3LYP,^42,43^ each augmented with D3^44^ dispersion corrections, and scalar-relativistic all-electron ZORA STO-TZ2P basis sets, all as implemented in the ADF 2019 program system.^45^ Point-group symmetry was used as appropriate, while all optimized structures were confirmed as minima via frequency analyses.

## Data Availability

Data Availability Statement: The data underlying this study are available in the published article and its online Supporting Information.

## Supporting Information Available

Optimized B3LYP-D3/ZORA-STO-TZ2P coordinates (PDF)
