# Supplementary material for: Theoretical Photoelectron Spectroscopy of Low-Valent Carbon Species: A ∼6 eV Range of Ionization Potentials among Carbenes, Ylides, and Carbodiphosphoranes
Source: ACS Org Inorg Au. 2022 Dec 2;3(2):92–5. doi: 10.1021/acsorginorgau.2c00045 (PMC10080723; doi:10.1021/acsorginorgau.2c00045)
Supplement: Supplementary file 1 — gg2c00045_si_001.pdf [file gg2c00045_si_001.pdf]

Supporting Information

Theoretical Photoelectron Spectroscopy of Low-Valent Carbon Species: A ~6-eV Range of Ionization Potentials Among Carbenes, Ylides, and Carbodiphosphoranes

Abhik Ghosh*[,a] and Jeanet Conradie*[,a,b]

[a]Department of Chemistry, University of Tromsø, N-9037 Tromsø, Norway;

[b]Department of Chemistry, University of the Free State, 9300 Bloemfontein, Republic of South Africa.

All-electron B3LYP-D3/ZORA-STO-TZ2P optimized Cartesian coordinates (Å)

Neutral molecules 2

1. $C(PMe_3)_2$, C_{2v}, q = 0, S = 0 2
2. $C(PPh_3)_2$, C_2, q = 0, S = 0 2
3. Me_2Im, C_{2v}, q = 0, S = 0 4
4. CF_2, C_{2v}, q = 0, S = 0 4
5. CCl_2, C_{2v}, q = 0, S = 0 4
6. $C\{P(NMe_2)_3\}_2$, C_1, q = 0, S = 0 4
7. CH_2PMe_3, C_s, q = 0, S = 0 6
8. CH_2SMe_2, C_s, q = 0, S = 0 6
9. carbodicarbene, C_2, q = 0, S = 0 6

Adiabatically ionized cations 7

10. $[C(PMe_3)_2]^+$, C_{2v}, q = 1, S = ½ 7
11. $[C(PPh_3)_2]^+$, C_2, q = 1, S = ½ 7
12. $[Me_2Im]^+$, C_{2v}, q = 1, S = ½ 9
13. $[CF_2]^+$, C_{2v}, q = 1, S = ½ 9
14. $[CCl_2]^+$, C_{2v}, q = 1, S = ½ 9
15. $[C\{P(NMe_2)_3\}_2]^+$, C_1, q = 1, S = ½ 9
16. $[CH_2PMe_3]^+$, C_s, q = 1, S = ½ 11
17. $[CH_2SMe_2]^+$, C_s, q = 1, S = ½ 11
18. carbodicarbene, C_2, q = 1, S = ½ 11

Neutral molecules

1. C(PMe$_3$)$_2$, C_{2v}, q = 0, S = 0

C	0.000000000	0.000000000	-0.664639000
C	2.031947000	1.420612000	0.990786000
C	2.031947000	-1.420612000	0.990786000
C	2.782359000	0.000000000	-1.404310000
C	-2.031947000	1.420612000	0.990786000
C	-2.031947000	-1.420612000	0.990786000
C	-2.782359000	0.000000000	-1.404310000
H	1.422087000	1.430892000	1.893909000
H	1.422087000	-1.430892000	1.893909000
H	1.858277000	2.345305000	0.442518000
H	1.858277000	-2.345305000	0.442518000
H	2.253194000	0.000000000	-2.353301000
H	3.085004000	1.355090000	1.269913000
H	3.085004000	-1.355090000	1.269913000
H	3.415142000	0.885077000	-1.343992000
H	3.415142000	-0.885077000	-1.343992000
H	-1.422087000	1.430892000	1.893909000
H	-1.422087000	-1.430892000	1.893909000
H	-1.858277000	2.345305000	0.442518000
H	-1.858277000	-2.345305000	0.442518000
H	-2.253194000	0.000000000	-2.353301000
H	-3.085004000	1.355090000	1.269913000
H	-3.085004000	-1.355090000	1.269913000
H	-3.415142000	0.885077000	-1.343992000
H	-3.415142000	-0.885077000	-1.343992000
P	1.515791000	0.000000000	-0.067600000
P	-1.515791000	0.000000000	-0.067600000

2. C(PPh$_3$)$_2$, C_2, q = 0, S = 0

C	0.000000000	0.000000000	1.036234000
C	0.176328000	2.421370000	-1.641053000
C	0.194759000	-2.166618000	-4.004923000
C	0.498902000	2.749261000	-2.948916000
C	0.852917000	-1.516273000	-1.367615000
C	0.873081000	-2.577445000	2.631418000
C	1.003496000	-3.732333000	3.391631000
C	1.206513000	-1.253185000	-3.742987000
C	1.277121000	-2.562755000	1.296900000
C	1.529710000	-4.887178000	2.823479000
C	1.535727000	-0.929669000	-2.432319000
C	1.801469000	-3.725642000	0.731574000
C	1.924384000	-4.882629000	1.490822000

C	2.823453000	-0.310220000	0.376685000
C	3.001191000	0.992158000	0.833843000
C	3.933452000	-1.041399000	-0.050048000
C	4.268602000	1.562171000	0.859504000
C	5.198860000	-0.472442000	-0.027037000
C	5.368395000	0.832008000	0.427716000
C	-0.176328000	-2.421370000	-1.641053000
C	-0.194759000	2.166618000	-4.004923000
C	-0.498902000	-2.749261000	-2.948916000
C	-0.852917000	1.516273000	-1.367615000
C	-0.873081000	2.577445000	2.631418000
C	-1.003496000	3.732333000	3.391631000
C	-1.206513000	1.253185000	-3.742987000
C	-1.277121000	2.562755000	1.296900000
C	-1.529710000	4.887178000	2.823479000
C	-1.535727000	0.929669000	-2.432319000
C	-1.801469000	3.725642000	0.731574000
C	-1.924384000	4.882629000	1.490822000
C	-2.823453000	0.310220000	0.376685000
C	-3.001191000	-0.992158000	0.833843000
C	-3.933452000	1.041399000	-0.050048000
C	-4.268602000	-1.562171000	0.859504000
C	-5.198860000	0.472442000	-0.027037000
C	-5.368395000	-0.832008000	0.427716000
H	0.058811000	2.419328000	-5.026110000
H	0.443668000	-1.677488000	3.051897000
H	0.688348000	-3.732732000	4.427162000
H	0.727693000	2.869485000	-0.824730000
H	1.292876000	3.458031000	-3.145487000
H	1.625198000	-5.789016000	3.414154000
H	1.737497000	-0.781755000	-4.559174000
H	2.094790000	-3.740965000	-0.309353000
H	2.132613000	1.540502000	1.175975000
H	2.320234000	-0.211194000	-2.242707000
H	2.323366000	-5.781812000	1.039534000
H	3.814907000	-2.056255000	-0.404032000
H	4.396683000	2.574830000	1.219713000
H	6.053771000	-1.046087000	-0.360925000
H	6.356327000	1.273507000	0.448594000
H	-0.058811000	-2.419328000	-5.026110000
H	-0.443668000	1.677488000	3.051897000
H	-0.688348000	3.732732000	4.427162000
H	-0.727693000	-2.869485000	-0.824730000
H	-1.292876000	-3.458031000	-3.145487000
H	-1.625198000	5.789016000	3.414154000
H	-1.737497000	0.781755000	-4.559174000
H	-2.094790000	3.740965000	-0.309353000
H	-2.132613000	-1.540502000	1.175975000
H	-2.320234000	0.211194000	-2.242707000
H	-2.323366000	5.781812000	1.039534000
H	-3.814907000	2.056255000	-0.404032000

H	-4.396683000	-2.574830000	1.219713000
H	-6.053771000	1.046087000	-0.360925000
H	-6.356327000	-1.273507000	0.448594000
P	1.112210000	-0.988774000	0.370956000
P	-1.112210000	0.988774000	0.370956000

3. Me_2Im, C_{2v}, q = 0, S = 0

C	0.000000000	0.000000000	0.973264000
C	0.000000000	0.675707000	-1.213131000
C	0.000000000	2.436937000	0.578755000
C	0.000000000	-0.675707000	-1.213131000
C	0.000000000	-2.436937000	0.578755000
H	0.000000000	1.375032000	-2.029545000
H	0.000000000	3.105820000	-0.279803000
H	0.000000000	-1.375032000	-2.029545000
H	0.000000000	-3.105820000	-0.279803000
H	0.884754000	2.630862000	1.182119000
H	0.884754000	-2.630862000	1.182119000
H	-0.884754000	2.630862000	1.182119000
H	-0.884754000	-2.630862000	1.182119000
N	0.000000000	1.058335000	0.119135000
N	0.000000000	-1.058335000	0.119135000

4. CF_2, C_{2v}, q = 0, S = 0

C	0.000000000	0.000000000	-0.730342000
F	1.034163000	0.000000000	0.066205000
F	-1.034163000	0.000000000	0.066205000

5. CCl_2, C_{2v}, q = 0, S = 0

C	0.000000000	0.000000000	-0.864581000
Cl	1.416686000	0.000000000	0.133324000
Cl	-1.416686000	0.000000000	0.133324000

6. $C\{P(NMe_2)_3\}_2$, C_1, q = 0, S = 0

C	0.074876000	-2.675715000	-1.143637000
C	0.278504000	0.386025000	-0.213275000
C	1.654679000	-0.096065000	-4.042490000
C	2.159190000	-1.353243000	2.042263000
C	2.548365000	1.007001000	2.337525000
C	2.818183000	0.386109000	-1.954888000
C	-0.544070000	1.895759000	3.506235000
C	-0.672138000	2.198553000	-3.024144000
C	-0.818372000	-2.133540000	-3.326048000

C	-0.956939000	-1.356647000	3.346583000
C	-0.975660000	2.773407000	1.273789000
C	-2.084201000	-1.253727000	1.208016000
C	-2.398599000	0.673476000	-2.315516000
H	0.025927000	-2.592138000	-3.856532000
H	0.040393000	1.162448000	4.054174000
H	0.279191000	-2.276001000	-0.156172000
H	0.371782000	2.289224000	-3.309802000
H	0.675614000	-0.090213000	-4.512422000
H	0.995603000	-3.126208000	-1.539262000
H	1.413366000	-2.142955000	2.073241000
H	2.079325000	1.969971000	2.517119000
H	2.123527000	-1.071709000	-4.222051000
H	2.268546000	0.662226000	-4.539149000
H	2.636148000	0.658392000	-0.919477000
H	2.654709000	-1.387550000	1.062869000
H	2.906204000	-1.554081000	2.812603000
H	3.117963000	1.073855000	1.401860000
H	3.250167000	0.803639000	3.149102000
H	3.390204000	1.184474000	-2.439436000
H	3.425981000	-0.527855000	-1.981534000
H	-0.017437000	-1.174953000	3.859290000
H	-0.208464000	2.890615000	3.816384000
H	-0.591111000	3.755416000	1.568813000
H	-0.671226000	-3.469407000	-1.048176000
H	-0.740535000	2.600184000	0.227786000
H	-0.850999000	2.862915000	-2.169017000
H	-1.190579000	-2.422497000	3.425592000
H	-1.220247000	-1.335317000	-3.942447000
H	-1.292514000	2.540299000	-3.855495000
H	-1.590723000	-2.899646000	-3.210071000
H	-1.596548000	1.795240000	3.799934000
H	-1.750416000	-0.804711000	3.866554000
H	-1.948416000	-1.032356000	0.154840000
H	-2.066795000	2.788148000	1.396188000
H	-2.347609000	-2.310284000	1.308202000
H	-2.612648000	1.224961000	-1.390349000
H	-2.652243000	-0.370865000	-2.154601000
H	-2.927407000	-0.659796000	1.586970000
H	-3.041212000	1.066212000	-3.105986000
N	1.542157000	-0.052533000	2.299640000
N	1.544919000	0.209253000	-2.626687000
N	-0.354668000	1.735850000	2.075220000
N	-0.440269000	-1.626808000	-2.012979000
N	-0.853027000	-0.989208000	1.939971000
N	-1.000476000	0.806549000	-2.723232000
P	0.123628000	-0.038961000	-1.750289000
P	0.139840000	0.278965000	1.379098000

7. CH_2PMe_3, C_s, q = 0, S = 0

C	1.457617000	-0.926916000	0.000000000
C	-0.096553000	1.029984000	1.476996000
C	-0.096553000	1.029984000	-1.476996000
C	-1.462584000	-1.108414000	0.000000000
H	0.744092000	1.722688000	1.476862000
H	0.744092000	1.722688000	-1.476862000
H	1.910466000	-1.240730000	0.930422000
H	1.910466000	-1.240730000	-0.930422000
H	-0.043883000	0.398993000	2.363926000
H	-0.043883000	0.398993000	-2.363926000
H	-1.029597000	1.592665000	1.492744000
H	-1.029597000	1.592665000	-1.492744000
H	-1.435686000	-1.738202000	0.888178000
H	-1.435686000	-1.738202000	-0.888178000
H	-2.378642000	-0.516681000	0.000000000
P	-0.039296000	0.003163000	0.000000000

8. CH_2SMe_2, C_s, q = 0, S = 0

C	0.086760000	0.975368000	1.572050000
C	0.086760000	0.975368000	-1.572050000
C	-0.617472000	-1.641390000	0.000000000
H	1.103727000	1.369371000	1.624299000
H	1.103727000	1.369371000	-1.624299000
H	-0.081059000	0.339918000	2.441287000
H	-0.081059000	0.339918000	-2.441287000
H	-0.596359000	1.825231000	1.628132000
H	-0.596359000	1.825231000	-1.628132000
H	-0.766009000	-2.199692000	0.914604000
H	-0.766009000	-2.199692000	-0.914604000
Si	-0.177155000	0.000743000	0.000000000

9. carbodicarbene, C_2, q = 0, S = 0

C	0.000000000	0.000000000	1.064595000
C	0.473563000	1.091444000	0.417946000
C	0.754220000	2.870235000	-1.023040000
C	1.476246000	3.130602000	0.081452000
C	-0.473563000	-1.091444000	0.417946000
C	-0.754220000	-2.870235000	-1.023040000
C	-1.476246000	-3.130602000	0.081452000
H	0.572534000	-1.248275000	-1.404417000
H	0.618982000	3.455659000	-1.913822000
H	1.663581000	1.982411000	1.882157000
H	2.090598000	3.979434000	0.318649000
H	-0.572534000	1.248275000	-1.404417000
H	-0.618982000	-3.455659000	-1.913822000

H	-1.663581000	-1.982411000	1.882157000
H	-2.090598000	-3.979434000	0.318649000
N	0.191403000	1.605759000	-0.859841000
N	1.336361000	2.047894000	0.935628000
N	-0.191403000	-1.605759000	-0.859841000
N	-1.336361000	-2.047894000	0.935628000

Adiabatically ionized cations

10. $[C(PMe_3)_2]^+$, C_{2v}, q = 1, S = ½

C	0.000000000	0.000000000	-0.668407000
C	1.932822000	1.459120000	0.981762000
C	1.932822000	-1.459120000	0.981762000
C	2.813795000	0.000000000	-1.380008000
C	-1.932822000	1.459120000	0.981762000
C	-1.932822000	-1.459120000	0.981762000
C	-2.813795000	0.000000000	-1.380008000
H	1.322756000	1.439851000	1.882371000
H	1.322756000	-1.439851000	1.882371000
H	1.714357000	2.361729000	0.413474000
H	1.714357000	-2.361729000	0.413474000
H	2.288770000	0.000000000	-2.331794000
H	2.987379000	1.460479000	1.257258000
H	2.987379000	-1.460479000	1.257258000
H	3.444061000	0.885647000	-1.315896000
H	3.444061000	-0.885647000	-1.315896000
H	-1.322756000	1.439851000	1.882371000
H	-1.322756000	-1.439851000	1.882371000
H	-1.714357000	2.361729000	0.413474000
H	-1.714357000	-2.361729000	0.413474000
H	-2.288770000	0.000000000	-2.331794000
H	-2.987379000	1.460479000	1.257258000
H	-2.987379000	-1.460479000	1.257258000
H	-3.444061000	0.885647000	-1.315896000
H	-3.444061000	-0.885647000	-1.315896000
P	1.577645000	0.000000000	-0.043195000
P	-1.577645000	0.000000000	-0.043195000

11. $[C(PPh_3)_2]^+$, C_2, q = 1, S = ½

C	0.000000000	0.000000000	1.067447000
C	0.129802000	-2.165883000	-4.016168000
C	0.239726000	2.409850000	-1.652035000
C	0.577045000	2.727894000	-2.958364000
C	0.813878000	-1.526731000	-1.393645000
C	0.833448000	-2.559342000	2.630103000
C	0.943607000	-3.715192000	3.390702000
C	1.168257000	-1.279164000	-3.764352000

C	1.198565000	-2.577750000	1.282101000
C	1.419917000	-4.887329000	2.815896000
C	1.510465000	-0.955625000	-2.458407000
C	1.674630000	-3.758308000	0.705388000
C	1.785075000	-4.907895000	1.474245000
C	2.759726000	-0.247309000	0.384053000
C	2.884495000	1.064690000	0.839673000
C	3.892829000	-0.955197000	-0.022589000
C	4.134427000	1.668314000	0.875480000
C	5.138172000	-0.346796000	0.016957000
C	5.259461000	0.965098000	0.463685000
C	-0.129802000	2.165883000	-4.016168000
C	-0.239726000	-2.409850000	-1.652035000
C	-0.577045000	-2.727894000	-2.958364000
C	-0.813878000	1.526731000	-1.393645000
C	-0.833448000	2.559342000	2.630103000
C	-0.943607000	3.715192000	3.390702000
C	-1.168257000	1.279164000	-3.764352000
C	-1.198565000	2.577750000	1.282101000
C	-1.419917000	4.887329000	2.815896000
C	-1.510465000	0.955625000	-2.458407000
C	-1.674630000	3.758308000	0.705388000
C	-1.785075000	4.907895000	1.474245000
C	-2.759726000	0.247309000	0.384053000
C	-2.884495000	-1.064690000	0.839673000
C	-3.892829000	0.955197000	-0.022589000
C	-4.134427000	-1.668314000	0.875480000
C	-5.138172000	0.346796000	0.016957000
C	-5.259461000	-0.965098000	0.463685000
H	0.129709000	2.420176000	-5.034924000
H	0.463865000	-1.644236000	3.070450000
H	0.658023000	-3.699271000	4.433674000
H	0.789428000	2.857936000	-0.835123000
H	1.386346000	3.419062000	-3.150817000
H	1.505142000	-5.786049000	3.411734000
H	1.714880000	-0.835327000	-4.584949000
H	1.946638000	-3.788526000	-0.340541000
H	2.013490000	1.605871000	1.183340000
H	2.152866000	-5.820231000	1.024970000
H	2.315422000	-0.259163000	-2.274123000
H	3.808086000	-1.977152000	-0.366736000
H	4.229408000	2.684822000	1.232415000
H	6.014683000	-0.897062000	-0.296542000
H	6.232824000	1.435457000	0.496498000
H	-0.129709000	-2.420176000	-5.034924000
H	-0.463865000	1.644236000	3.070450000
H	-0.658023000	3.699271000	4.433674000
H	-0.789428000	-2.857936000	-0.835123000
H	-1.386346000	-3.419062000	-3.150817000
H	-1.505142000	5.786049000	3.411734000
H	-1.714880000	0.835327000	-4.584949000

H	-1.946638000	3.788526000	-0.340541000
H	-2.013490000	-1.605871000	1.183340000
H	-2.152866000	5.820231000	1.024970000
H	-2.315422000	0.259163000	-2.274123000
H	-3.808086000	1.977152000	-0.366736000
H	-4.229408000	-2.684822000	1.232415000
H	-6.014683000	0.897062000	-0.296542000
H	-6.232824000	-1.435457000	0.496498000
P	1.134249000	-1.042631000	0.326486000
P	-1.134249000	1.042631000	0.326486000

12. $[Me_2Im]^+$, C_{2v}, q = 1, S = ½

C	0.000000000	0.000000000	0.961912000
C	0.000000000	0.713098000	-1.197750000
C	0.000000000	2.466944000	0.580029000
C	0.000000000	-0.713098000	-1.197750000
C	0.000000000	-2.466944000	0.580029000
H	0.000000000	1.386474000	-2.040925000
H	0.000000000	3.159107000	-0.256532000
H	0.000000000	-1.386474000	-2.040925000
H	0.000000000	-3.159107000	-0.256532000
H	0.886368000	2.611594000	1.193124000
H	0.886368000	-2.611594000	1.193124000
H	-0.886368000	2.611594000	1.193124000
H	-0.886368000	-2.611594000	1.193124000
N	0.000000000	1.088817000	0.074255000
N	0.000000000	-1.088817000	0.074255000

13. $[CF_2]^+$, C_{2v}, q = 1, S = ½

C	0.000000000	0.000000000	-0.576130000
F	1.083866000	0.000000000	-0.010901000
F	-1.083866000	0.000000000	-0.010901000

14. $[CCl_2]^+$, C_{2v}, q = 1, S = ½

C	0.000000000	0.000000000	-0.598537000
Cl	1.478278000	0.000000000	0.000303000
Cl	-1.478278000	0.000000000	0.000303000

15. $[C\{P(NMe_2)_3\}_2]^+$, C_1, q = 1, S = ½

C	0.309068000	0.343344000	-0.204030000
C	1.856185000	-0.548516000	-3.842279000
C	2.294808000	-1.310071000	1.941386000
C	2.453519000	1.077203000	2.441718000

C	2.632435000	0.987349000	-2.102717000
C	-0.010631000	-2.724178000	-1.266961000
C	-0.338876000	2.915863000	1.336892000
C	-0.548503000	2.215692000	-3.016235000
C	-0.711035000	-1.698930000	3.288060000
C	-1.175183000	1.721180000	3.302212000
C	-1.214977000	-2.023305000	-3.281618000
C	-2.047162000	-1.389772000	1.257332000
C	-2.390209000	0.860867000	-2.152419000
H	0.144749000	-1.311467000	3.831394000
H	0.173556000	2.778827000	0.390509000
H	0.258038000	3.571554000	1.977098000
H	0.382396000	-2.361705000	-0.320724000
H	0.470787000	2.177447000	-3.388097000
H	0.774942000	-3.280934000	-1.785652000
H	1.141481000	-1.332507000	-4.066444000
H	1.600180000	-2.131086000	1.788421000
H	1.803857000	0.204680000	-4.633917000
H	1.879529000	1.955468000	2.721276000
H	2.357630000	1.433510000	-1.152748000
H	2.768687000	1.778917000	-2.845215000
H	2.854801000	-0.987176000	-3.852551000
H	2.919999000	-1.547251000	2.802570000
H	2.937614000	-1.223571000	1.060010000
H	3.062493000	1.315930000	1.563936000
H	3.121910000	0.834991000	3.268054000
H	3.582598000	0.462556000	-1.987317000
H	-0.501234000	2.201961000	4.017173000
H	-0.598987000	-2.779998000	3.181571000
H	-0.606591000	2.962894000	-2.218413000
H	-0.626515000	-2.742665000	-3.857550000
H	-0.834171000	-3.407175000	-1.053424000
H	-1.193601000	2.536525000	-3.834478000
H	-1.298826000	3.402553000	1.153607000
H	-1.432092000	-1.167276000	-3.912251000
H	-1.443461000	0.743388000	3.686361000
H	-1.613320000	-1.509462000	3.875701000
H	-1.996696000	-1.028179000	0.234001000
H	-2.084498000	2.319684000	3.233001000
H	-2.156420000	-2.503672000	-3.006371000
H	-2.187092000	-2.471341000	1.228866000
H	-2.581490000	1.541157000	-1.316874000
H	-2.679129000	-0.143943000	-1.858582000
H	-2.915948000	-0.941690000	1.747915000
H	-3.015324000	1.163428000	-2.993019000
N	1.567954000	-0.063723000	2.203571000
N	1.599127000	0.042383000	-2.528987000
N	-0.500260000	-1.608568000	-2.073096000
N	-0.560230000	1.619006000	1.978760000
N	-0.808291000	-1.077592000	1.965565000
N	-0.983593000	0.893556000	-2.564280000

P	0.106338000	0.203724000	1.439418000
P	0.123106000	-0.095710000	-1.795433000

16. $[CH_2PMe_3]^+$, C_s, q = 1, S = ½

C	1.457617000	-0.926916000	0.000000000
C	-0.096553000	1.029984000	1.476996000
C	-0.096553000	1.029984000	-1.476996000
C	-1.462584000	-1.108414000	0.000000000
H	0.744092000	1.722688000	1.476862000
H	0.744092000	1.722688000	-1.476862000
H	1.910466000	-1.240730000	0.930422000
H	1.910466000	-1.240730000	-0.930422000
H	-0.043883000	0.398993000	2.363926000
H	-0.043883000	0.398993000	-2.363926000
H	-1.029597000	1.592665000	1.492744000
H	-1.029597000	1.592665000	-1.492744000
H	-1.435686000	-1.738202000	0.888178000
H	-1.435686000	-1.738202000	-0.888178000
H	-2.378642000	-0.516681000	0.000000000
P	-0.039296000	0.003163000	0.000000000

17. $[CH_2SMe_2]^+$, C_s, q = 1, S = ½

C	0.079908000	0.965983000	1.591561000
C	0.079908000	0.965983000	-1.591561000
C	-0.611209000	-1.648149000	0.000000000
H	1.104944000	1.345250000	1.654296000
H	1.104944000	1.345250000	-1.654296000
H	-0.115246000	0.323812000	2.448954000
H	-0.115246000	0.323812000	-2.448954000
H	-0.578601000	1.838700000	1.637102000
H	-0.578601000	1.838700000	-1.637102000
H	-0.758644000	-2.207741000	0.916861000
H	-0.758644000	-2.207741000	-0.916861000
Si	-0.154023000	0.095886000	0.000000000

18. carbodicarbene, C_2, q = 1, S = ½

C	0.000000000	0.000000000	1.009953000
C	0.449426000	1.135559000	0.372632000
C	0.712501000	2.932381000	-0.989823000
C	1.501229000	3.121498000	0.099521000
C	-0.449426000	-1.135559000	0.372632000
C	-0.712501000	-2.932381000	-0.989823000
C	-1.501229000	-3.121498000	0.099521000
H	0.540412000	3.565504000	-1.840944000
H	0.609893000	-1.337174000	-1.438910000

H	1.733247000	1.892956000	1.825794000
H	2.157622000	3.935584000	0.346735000
H	-0.540412000	-3.565504000	-1.840944000
H	-0.609893000	1.337174000	-1.438910000
H	-1.733247000	-1.892956000	1.825794000
H	-2.157622000	-3.935584000	0.346735000
N	0.098701000	1.712384000	-0.830555000
N	1.319688000	2.038964000	0.917585000
N	-0.098701000	-1.712384000	-0.830555000
N	-1.319688000	-2.038964000	0.917585000
